# Characterizing the Cauline Domatia of Two Newly Discovered Ecuadorian Ant Plants in *Piper*: An Example of Convergent Evolution

**DOI:** 10.1673/031.009.2701

**Published:** 2009-06-02

**Authors:** Eric J. Tepe, Walter A. Kelley, Genoveva Rodriguez-Castañeda, Lee A. Dyer

**Affiliations:** ^1^ Department of Biology, University of Utah, Salt Lake City, UT; ^2^ Department of Biology, Mesa State College, Grand Junction, CO; ^3^ Biology Department, University of Nevada, Reno, NV

**Keywords:** myrmecophytes, pearl bodies, Piperaceae, *Pheidole*, insect-plant mutualism

## Abstract

The stems of some myrmecophytes in *Piper* are used as domatia by resident ant colonies. Hollow, ant-occupied stems were previously known only in four species of southern Central American *Piper*, all members of Section *Macrostachys*. Here we present two additional, unrelated, hollow-stemmed myrmecophytes from Ecuador: *P. immutatum* and *P. pterocladum* (members of sections *Radula* and *Peltobryon*, respectively). Although similar superficially, stem cavities of the Ecuadorian *Piper* species differ morphologically and developmentally from those of Central American taxa. The stem cavities of *P. immutatum*, and possibly *P. pterocladum*, are formed during stem development, and begin forming only a few millimeters behind the apical meristem. This mode of cavity formation differs markedly from myrmecophytes in section *Macrostachys*, where the stems remain solid unless excavated by the specialized ant partner *Pheidole bicornis*. The stems of *P. immutatum* and *P. pterocladum* do not produce wound-response tissue around the cavity, unlike the stems in section *Macrostachys*. The entrance holes in stems of *P. immutatum* are formed through apoptotic processes and are located at each node below the petiole, whereas those in section *Macrostachys* are excavated by the ants in the leaf axil. This study documents convergent evolution of ant-plant associations in *Piper*, and emphasizes the need for careful comparison of apparently homologous, ant-associated structures in specialized myrmecophytes.

## Introduction

Hollow stems of some neotropical *Piper* species are used as domatia by specialized ant residents. Until now, stem dwelling ants in *Piper* have been reported only from a small complex of five *Piper* species centered in Costa Rica (Risch et al. 1977; Letourneau 1983; Tepe et al. 2004). These myrmecophytic associations, first described by Burger (1971, 1972), have been the focus of many ecological and evolutionary studies (Risch 1982; Letourneau 1998; Dyer and Letourneau 1999; Fischer et al. 2002, 2003; Dyer et al. 2003; Dyer and Palmer 2004; Tepe et al. 2004, 2007a, b). While these are the only formally described relationships between *Piper* and ants, a number of anecdotal reports imply facultative relationships between these groups throughout the tropics. Nevertheless, in a pantropical genus of ca. 2000 species (Quijano-Abril et al. 2006), it is noteworthy that so few *Piper* myrmecophytes are known. We have recently discovered well developed *Piper* myrmecophytes in eastern Ecuador. Examination of the stems of these myrmecophytes reveals that, although superficially similar, they differ in almost all morphological and developmental characters from the stems in Central American myrmecophytes (Tepe et al. 2007a).

*Piper immutatum* Trel. is found on the eastern slopes of the Andes in Ecuador and Peru (EJT, pers. obs.). It typically grows as a small, unbranched plant, ranging in height from 20 cm to 2.5 m in the rain forest understory around moist quebradas. Larger, branched plants are sometimes found near the forest edge, but only in shaded areas. *Piper immutatum* is not widespread, but is locally abundant in favorable habitats. *Piper pterocladum* C. DC. is restricted to Ecuador and occurs in similar habitats as *P. immutatum*, but is much less common. *Piper pterocladum* reaches 3 m in height and grows as a slender, single-stemmed, rarely branched plant. The older stems accumulate little wood, with the stems remaining slender, even in large individuals.

This study characterizes the stem cavities of *P. immutatum* and *P. pterocladum* and compares them to the cavities in the previously known Central American myrmecophytes in *Piper* section *Macrostachys* (Tepe et al. 2007a). Our goal is to better understand the diversity of plant characters that support ant-plant associations in *Piper*. Characterizing such relationships is the first step in a process that will contribute to studies of the ecology and evolution of multi-trophic interactions. Myrmecophytes have proven to be a model system for studies of trophic interactions in the tropics (Dyer 2008).

## Materials and Methods

Material for this study was collected and observations of the ant-plant relationships were made on the eastern slopes of the Ecuadorian Andes, at elevations ranging between 400 and 1500 m. Stems and petioles were preserved in 70% ethanol, and herbarium vouchers were made for all accessions. Vouchers were deposited at the Herbario Nacional del Ecuador (QCNE) and the W. S. Turrell Herbarium at Miami University (MU). Stems were sectioned using a Vibratome (Series-1000, Vibratome, St. Louis, Missouri, USA), stained with safranin-fast green or toluidine blue, examined using standard light and dissecting microscopy, and imaged with a SPOT digital camera (Diagnostic Instruments, Inc., Sterling Heights, Michigan, USA). Unless otherwise specified, all sections were taken from the third youngest internode, half way between the adjacent nodes. Observations of stem anatomy are based on examination of five individuals from each of four populations of *P. immutatum* (EJT 1590, 1601, 1611, 1632), and three individuals from one population of *P. pterocladum* (EJT 1610). Additionally, 100 individuals of *P. immutatum* and 60 of *P. pterocladum* were destructively sampled to determine colony size of the ant inhabitants.

*Piper immutatum* was grown from seed in the greenhouses at Mesa State College (Grand Junction, Colorado, USA) in the absence of its stem inhabiting ant partner, *Pheidole* sp. Observations were recorded for all stages of plant development for 15 individuals, from seed germination to maturity.

## Results and Discussion

**Figure 1.**
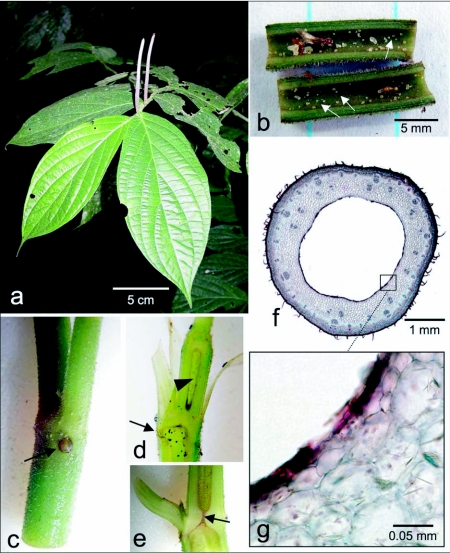
*Piper immutatum*: (a) *Piper immutatum* habit. (b) Longitudinal section through a stem showing pearl body production on the walls of the stem cavity (arrows). Note the presence of several castes and several generations of ant residents are also present. (c) Photo of stem showing the formation of the entrance hole (arrow) below the petiole. Compare Figs. 1 c–e with 1 b for scale, (d) Longitudinal section through the apical portion of a stem showing the developing stem cavities (arrowhead), and the entrance hole (arrow). (e) Longitudinal section through a node. The arrow indicates the hole through the node that connects the internodal chambers. (f) Cross section through a stem with a fully developed stem cavity. (g) Close-up of the cavity wall showing living cells (transparent), and cell wall remains of lysed cells (red).

**Figure 2.**
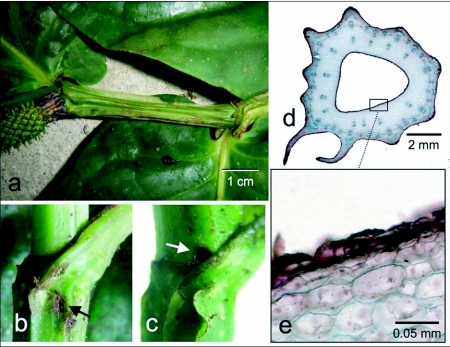
*Piper pterocladum*: (a) habit; note the winged stems characteristic of this species. (b, c) nodes showing the variable location of the entrance hole in this species (arrows): below the petiole in b, and above the petiole in c. Compare to Figure 2a for scale. (d) Cross section through the stem showing the stem cavity. (e) Close-up of the cavity wall showing living cells (transparent), and cell wall remains of lysed cells (red).

Both *P. immutatum* (Figure 1a) and *P. pterocladum* (Figure 2a) are occupied by the same unnamed species of *Pheidole* (J. Longino, Evergreen State College, personal communication). Based on our observations thus far, both plant species are always found with hollow stems, and occupation rates are close to 100%. Petioles of both species are terete and do not form domatia. Pearl bodies are produced in abundance on the inner surface of the stem cavities (Figure 1b). Pearl bodies are single cells that swell with lipids, proteins, and carbohydrates, and appear to be the primary, if not the sole source of nutrition for the ants in Central American myrmecophytes (Rickson and Risch 1984; Fischer et al. 2002). Presumably, ants eat the pearl bodies in the Ecuadorian *Piper* species as well. The ant colonies that inhabit *P. immutatum* and *P. pterocladum* are similar to those that inhabit the Costa Rican myrmecophytes in that they are relatively small, with an average of 45 major workers; the ants are not aggressive (relative to the fierce plant ants *Pseudomyrmex* and *Azteca*); their sting is weak; and they do not respond in large numbers when the plants are disturbed. The precise benefit of the ants to the plants is currently under study. The ants gain access to the stem cavity through entrance holes found at each node. The stem cavities are 3.5–4 mm in diameter, and the ants occupy the entire plant from ground level to the youngest shoots, in which the cavities are always fully formed. The ants occupy plants of all sizes and colony size increases as plant size increases.

### Piper immutatum

The entrance holes to the stem cavity are located at each node below the point of petiole insertion (Figure 1c). Observations of greenhouse-grown plants reveals that the entrance holes form naturally during stem maturation during plant development. The entrance holes in most myrmecophytes are formed spontaneously, or are excavated through natural gaps in the vascular tissue (Bailey 1922; Federle et al. 2001; Moog et al. 2002). However, the location in *P. immutatum* is unusual since no natural break in the vascular tissue is found in this part of the stem. Initially, the holes are large enough for a colonizing queen to enter, but later the holes grow closed due to the accumulation of callus in unoccupied plants.

Similarly, the stem cavities also form during plant development (Figure 1d). The cavities are lysigenous in origin and are formed only a few millimeters behind the apical meristem. As the internodes become hollow, a small hole 0.5–1.0 mm in diameter forms in the center of the solid node, creating continuous stem cavities (Figure 1e). The first 3–5 internodes of the seedling are short and solid; the following internodes are longer and all are hollow. Thus, a continuous stem cavity extends from near the base of the plant up to near the apical meristem. Microscopy reveals that the stem cavity is lined with fragments of the cell walls that ruptured during cavity development, but the stem does not produce wound response tissue (Figures 1f, 1g). Pearl bodies are produced by the layer of cells lining the cavity (Figure 1b). Pearl body production appears to be distributed more or less evenly throughout the stem cavities; however, ant activity is concentrated in younger parts of the stem. Based on greenhouse observations, it appears that pearl bodies are produced only when the stem cavities are occupied by an organism. So far, ants, nematodes, and aphids have been found to elicit pearl body production.

### Piper pterocladum

The ant-associated characters of *P. pterocladum* are similar to those of *P. immutatum*. However, unlike *P. immutatum*, the entrance holes are variable in their position and may be located above or below the petiole at a given node (Figures 2b, 2c). No wound response tissue is formed on the walls of the stem cavity (Figures 2d, 2e), and pearl bodies are produced on these walls. The development of the stem cavities and entrance holes remains unstudied because we have not yet been able to grow this species in the greenhouse. The variable position of the entrance holes suggests that they are excavated by the ants. Much remains to be learned about the development of these structures in *P. pterocladum*.

## Comparison between Ecuadorian and Central American myrmecophytes

**Figure 3.**
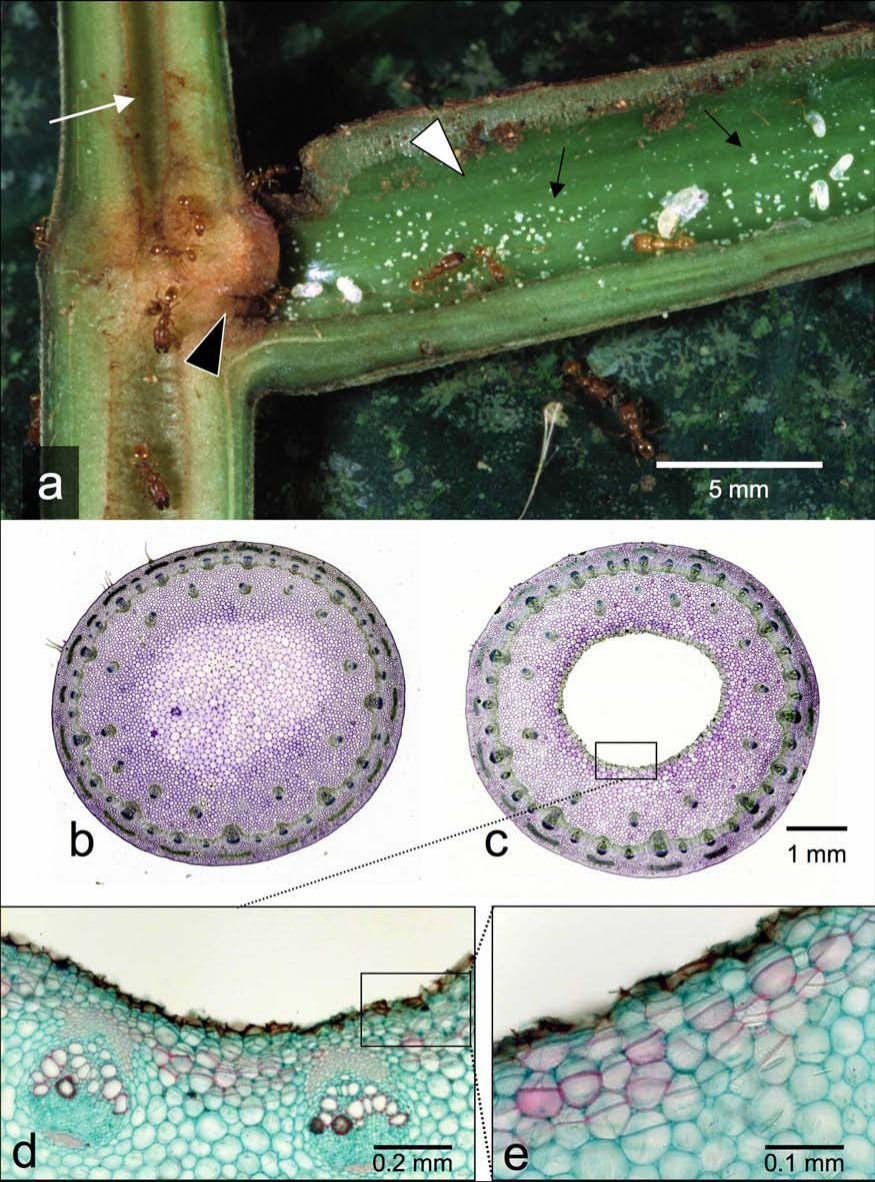
The Central American myrmecophytes of *Piper* section *Macrostachys*. (a) Longitudinal section through the stem and petiole of *P. cenocladum*. The chamber formed by the sheathing petiole forms the primary domatium in these species (white arrowhead). Note the excavated domatium in the stem cavity (white arrow) and the passage between petiolar and cauline domatia (black arrowhead). Pearl bodies are produced in the petiolar chamber (black arrows). The larger white structures are ant larvae and pupae, and two castes of workers are visible. Photo by Greg Dimijian. (b) Cross section of an unexcavated stem of *P. sagittifolium* from the youngest internode. (c) Cross section of an older, excavated portion of the same stem (third youngest internode). (d) Close up of the wall of the stem cavity showing the well-defined wound response layer. (e) Close up of the wound response later. Note the additional cell divisions and the differentiated cell walls in this section (indicated by the difference in color).

Many aspects of the ant-plant associations found in *P. immutatum* and *P. pterocladum* are similar to those in section *Macrostachys*. Both sets of myrmecophytes are occupied by small colonies of *Pheidole* species that are not particularly aggressive. However, the most obvious difference between the sets of myrmecophytes is the nature of the domatia that are preformed by the plant. In section *Macrostachys*, the domatia formed by the sheathing petioles are available for immediate occupation (Figure 3a) (Tepe et al. 2007b), but the stems remain solid until excavated by ant residents (Figures 3b and c; Tepe et al. 2007a). In *P. immutatum*, on the other hand, the petioles of *P. immutatum* and *P. pterocladum* are terete and do not form closed chambers, but the stem cavities and entrance holes form during stem development and are available without modification for habitation by a founding queen. Consequently, the location of pearl body production differs between the two sets of myrmecophytes: Pearl bodies are produced on the surface of the stem cavity in Ecuadorian myrmecophytes; in section *Macrostachys*, they are produced primarily inside the sheathing petiole chambers and are never found in the stem cavity (Figure 3a; Risch et al. 1977) (Tepe et al. 2007b).

The most striking differences between Ecuadorian and Central American myrmecophytes are the anatomical and developmental variances in their stem cavities. Once excavated, the stems of section *Macrostachys* produce a layer of wound response tissue on the cavity walls (Figures 3d, 3e). This layer is suberized and presumably isolates the living plant body from the cavity. Fischer et al. (2003) demonstrated that several *Piper* species in section *Macrostachys* are able to absorb nutrients from their ant occupants, mostly in younger parts of the stems. However, no evidence of nutrient absorption was observed when supplemental nutrients were injected into the cavities of older parts of the stems (Letourneau, 1998). The cavities of the Ecuadorian species are not surrounded by specialized tissue and the cavity is lined by living cells. This fundamental difference between the two sets of myrmecophytes explains why pearl bodies can be produced in the stem cavities of the Ecuadorian species, but not in those of section *Macrostachys*. The wound response layer in section *Macrostachys* is made up of differentiated cells that are fixed in their morphology and dimensions; as a result, they cannot produce pearl bodies. In contrast, the cells that line the stem cavities in Ecuadorian species are undifferentiated parenchyma and can thus produce pearl bodies continuously.

Whereas the entrance holes in *P. immutatum* forms spontaneously during stem development, those in section *Macrostachys* are excavated by the ant residents. The location of the entrance holes differs as well. Entrance holes in *P. immutatum* are located below the petiole, whereas those in section *Macrostachys* are mainly found above the petioles. The development of the entrance holes in *P. pterocladum*, which vary in their location, requires additional study. If not maintained by ants, the entrance holes in all myrmecophytes eventually become sealed by an accumulation of callus.

## Evolution of myrmecophytism in *Piper*

**Figure 4.**
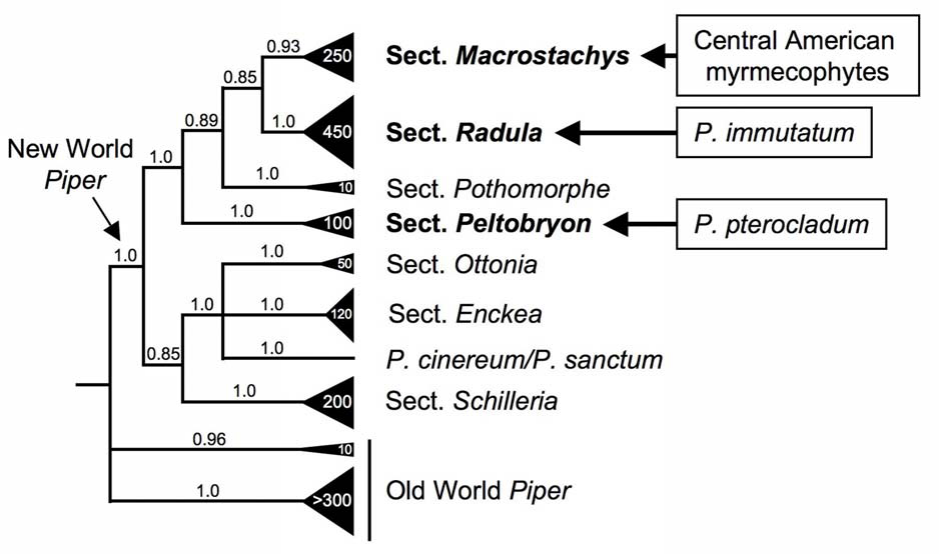
Summary phylogeny of the sections of *Piper* from Bayesian analysis of nuclear ITS and plastid *psbj-petA* sequences (redrawn from Jaramillo et al. 2008). Numbers above branches are Bayesian posterior probabilities, and numbers in the triangles indicate the approximate number of species in each section. Placement of the myrmecophytes within New World *Piper* are shown. Each myrmecophyte subgroup is nested within a clade of non-myrmecophytes, indicating at least three independent origins of ant-plant association in New World *Piper*.

All of the known myrmecophytes from Central America (five species) belong to *Piper* section *Macrostachys*. The two Ecuadorian species in this study represent two separate sections of *Piper* (Figure 4): *P. immutatum* is a member of *Piper* section *Radula*, while *P. pterocladum* belongs in section *Peltobryon*. These myrmecophytes are members of three separate clades of non-myrmecophytes (section *Macrostachys* = 200–250 species, section *Radula* = ca. 450 species, and section *Peltobryon* = ca. 50 species; Figure 4; Jaramillo et al. 2008). In each case, the myrmecophytes are relatively derived members of their respective clades; as a result, regardless of the relationships among the sections, *P. immutatum, P. pterocladum*, and the Central American myrmecophytes are not closely related to each other. Thus, the presence of myrmecophytes in these clades represents at least three independent origins of myrmecophytism and of plant characters that support ant-plant associations in *Piper* including domatia, entrance holes to the domatia, and year-round food body production in concealed locations.

We are currently aware of two centers of myrmecophytism among New World *Piper*: one in southern Central America and one in eastern Ecuador. In Central America, the distribution of myrmecophytism in *Piper* appears to be correlated with the range of *Pheidole bicornis*, the specialized ant partner. Interestingly, myrmecophytism is facultative; several of these plant species have broader distributions and occur without ants outside of this area of myrmecophytism. The identity and range of the undescribed South American ant species remain unknown, but within the large genus *Pheidole*, it is apparently not closely related to *P. bicornis*: the unnamed South American taxon belongs to the tribe *Scrobifera*, whereas *Pheidole bicornis* belongs to the tribe *Transversostriata* (*fide* Wilson 2003 as determined by GRC). Our findings thus suggest that *Pheidole* has adapted to living symbiotically with *Piper* at least twice independently. During a recent trip to Peru, the senior author collected a single individual of *Piper costatum*, a close relative of *P. pterocladum* that was occupied by a species of *Pheidole*, as well as another unidentified *Piper* species with hollow stems not occupied by ants. These discoveries imply that many additional ant-*Piper* associations remain to be discovered throughout Andean South America.

Nutrient uptake has been demonstrated in the Central American *Piper* myrmecophytes in which older stem cavities are lined with a wound response layer (Fischer et al., 2003); does the absence of a wound response layer in the stems of the South American species allow for greater nutrient absorption by the plants from frass, dead ants, and materials that the ants bring into the stem chamber? The stem chambers of the two species presented here are functionally similar to those of *Piper* section *Macrostachys*, but differ morphologically and developmentally. It is not clear whether the ants are taking advantage of existing hollow stems in the South American myrmecophytes (i.e. transfer exaptation; Arnold 1994), or if hollow stems evolved in association with the ants. Either way, it is remarkable that only eight hollow-stemmed *Piper* species are known (both excavated and naturally hollow) among the estimated 2000 species of *Piper* found worldwide (Quijano-Abril et al. 2006).

*Piper* myrmecophytes are members of three distantly related groups of *Piper*, yet similarities among the ant-plant associations are striking. Similarities among the two South American *Piper* species are especially remarkable; their stem cavities, although differing developmentally and anatomically, are functionally analogous and all of the myrmecophytes produce pearl bodies in large numbers. Pearl body production is widespread in the Piperaceae, occurring on young leaves, stems, and inflorescences. However, production in large numbers and in concealed locations is, as far as we know, restricted to myrmecophytes. All *Piper* myrmecophytes studied thus far are shade-tolerant species and all are occupied by species of *Pheidole*. Similarly, the *Pheidole* species that occupy Central and South American myrmecophytes are distantly related within the genus of >600 species (Wilson 2003), yet their behavior is similar and both utilize nutrients from plant tissues. *Piper-Pheidole* associations thus present an excellent example of convergent evolution.

In conclusion, the discovery of these two Ecuadorian myrmecophytes expands our understanding of *Piper* ant-plant associations. When more details are uncovered about the ants and plants, these two separate sets of *Piper-Pheidole* associations will allow us to explore the conditions necessary for the evolution of ant-plant associations in both *Piper* and *Pheidole*, and permit a comparison of factors that limit the distribution of myrmecophytes. Similarities in the associations between ants and plants from two geographically separated areas, representing three distantly related groups of *Piper*, represent a remarkable example of convergent evolution of a plant-animal mutualism. At the same time, they illustrate that the homology of ant-associated structures in plant myrmecophytes, however similar in appearance and function, should never be assumed.

## Editor's note

Paper copies of this article will be deposited in the following libraries. Senckenberg Library, Frankfurt Germany; National Museum of Natural History, Paris, France; Field Museum of Natural History, Chicago, Illinois USA; the University of Wisconsin, Madison, USA; the University of Arizona, Tucson, Arizona USA; Smithsonian Institution Libraries, Washington D.C. USA; The Linnean Society, London, England.
